# Assessment of Sociodemographics and Inflation-Related Stress in the US

**DOI:** 10.1001/jamanetworkopen.2023.13431

**Published:** 2023-05-15

**Authors:** Cary Wu, Patricia Louie, Alex Bierman, Scott Schieman

**Affiliations:** 1 York University, Toronto, Ontario, Canada; 2 University of Washington, Seattle; 3 University of Calgary, Calgary, Alberta, Canada; 4 University of Toronto, Toronto, Ontario, Canada

## Abstract

This survey study of US adults examines the association of stress experienced as a result of inflation with sociodemographic characteristics such as sex, race and ethnicity, education, and income levels.

## Introduction

The inflation rate in the US is higher than it has been since the early 1980s. High inflation can harm individuals’ well-being.^1,2^ The recent cycles of the Stress in America survey (March and October 2022) have reported that high inflation has become a significant source of stress for most American adults.^3,4^ Experiences of stress often produce damaging effects on health.^5^ However, less known is how inflation stress is distributed within the population. The unequal distribution of inflation stress may exacerbate inequalities in health. In this survey study, we consider how inflation stress varies across sociodemographic groups in the American population.

## Methods

We analyzed data from the Household Pulse Survey (HPS) collected from September 2022 to February 2023 by the US Census Bureau.^6^ The data are publicly available and therefore this study was exempt from ethical review and approval by the Research Ethics Committee at York University as well as the requirement for informed consent. We measured inflation stress using the survey question, “How stressful has the increase in prices in the last 2 months been for you?” Responses were coded on an ordered scale including not at all stressful, a little stressful, moderately stressful, and very stressful. Sociodemographic variables included gender, race and ethnicity, age, marital status, education, and household income. We estimated odds ratios (ORs) with 95% CIs of outcomes using ordinal logistic regressions. Following the American Association for Public Opinion Research (AAPOR) reporting guideline, we reported cross-tabulated results for transparency and used weights for representative population estimates. The eAppendix in Supplement 1 details our analytical strategy.

## Results

Of the 369 328 respondents, 18 923 (5.1%) were Asian, 41 274 (11.2%) were Black, 63 803 (17.3%) were Hispanic, and 229 407 (62.1%) were White. Respondents had a median age of 49 (range, 18-89 years), 189 389 (51.3%) were women, and 117 280 (31.8%) had a bachelor’s degree or higher. Among 322 507 respondents (93.2%) who reported that prices for goods and services have increased in the area where they live and shop in the last 2 months, 152 548 (47.3%) indicated that the increase in prices has been very stressful, 91 033 (28.2%) felt moderately stressful, 60 696 (18.9%) felt a little stressful, and only 17 957 (5.6%) felt that increases in prices were not at all stressful (Table 1).

**Table 1.** Summary Inflation Stress Levels by Sociodemographic Variables^a^

Characteristics	Respondents, No. (%) (N = 322 507)			
	Not at all stressful	A little stressful	Moderately stressful	Very stressful
Overall	17 957 (5.6)	60 969 (18.9)	91 033 (28.2)	152 548 (47.3)
Sex (at birth)				
Male	11 122 (7.2)	31 595 (20.3)	44 560 (28.7)	68 052 (43.8)
Female	6835 (4.1)	29 374 (17.6)	46 473 (27.8)	84 496 (50.5)
Race and ethnicity (self-reported)^b^				
Asian	962 (5.9)	3746 (22.9)	5233 (32.0)	64,30 (39.3)
Black	22,28 (6.4)	5532 (15.8)	8236 (23.6)	18 870 (54.1)
Hispanic	1801 (3.3)	7043 (13.0)	13 895 (25.7)	31 334 (58.0)
White	12 409 (6.1)	42 615 (21.0)	60 123 (29.6)	88 256 (43.4)
Other	556 (4.0)	2032 (14.7)	3546 (25.7)	76,58 (55.5)
Age group	1760 (3.3)	9300 (17.3)	16 991 (31.7)	25 638 (47.8)
18-30 y	2235 (3.9)	9881 (17.2)	15 247 (26.6)	30 022 (52.3)
31-40 y	2311 (4.3)	8903 (16.4)	14 212 (26.2)	28 844 (53.2)
41-50 y	2889 (5.2)	9835 (17.6)	14 820 (26.5)	28 495 (50.9)
51-60 y	4452 (7.3)	13 008 (21.4)	17 571 (29.0)	25 642 (42.3)
61-70 y	4309 (10.7)	10 043 (24.9)	12 193 (30.1)	13 906 (34.4)
≥71 y	17 957 (5.6)	60 969 (18.9)	91 033 (28.2)	15 2548 (47.3)
Marital status				
Married	11 641 (6.5)	37 337 (20.7)	51 450 (28.6)	79 579 (44.2)
Widowed	984 (6.4)	2763 (18.0)	4091 (26.7)	7500 (48.9)
Divorced	1737 (4.4)	5786 (14.7)	10 369 (26.3)	21 578 (54.7)
Separated	190 (2.8)	712 (10.6)	1433 (21.3)	4386 (65.4)
Never married	3357 (4.2)	14 193 (17.8)	23 402 (29.0)	38 842 (48.7)
Education				
Less than high school	282 (3.9)	821 (11.3)	1365 (18.8)	4804 (66.1)
Some high school	485 (3.2)	1705 (11.2)	3498 (22.9)	9587 (62.8)
High school	4037 (4.1)	13 490 (13.7)	25 659 (26.0)	55 492 (56.2)
Some college	2602 (3.9)	10 930 (16.2)	19 266 (28.6)	34 663 (51.4)
Associate’s	1224 (4.0)	4906 (16.2)	8628 (28.4)	15 618 (51.5)
Bachelor’s	4229 (7.4)	14 592 (25.6)	18 410 (32.2)	19 866 (34.8)
Graduate	5098 (11.0)	14 525 (31.3)	14 207 (30.7)	12 519 (27.0)
Household income				
<$25 000	965 (2.7)	3559 (9.9)	7666 (21.3)	23 872 (66.2)
$25 000-$34 999	727 (2.5)	3372 (11.5)	7119 (24.3)	18 111 (61.8)
$35 000-$49 999	975 (2.9)	4643 (13.8)	9071 (26.9)	19 003 (56.4)
$50 000-$74 999	1951 (4.1)	7864 (16.7)	14 052 (29.9)	23 191 (49.3)
$75 000-$99 999	1989 (5.7)	7307 (20.8)	10 995 (31.3)	14 847 (42.3)
$100 000-$149 999	2928 (7.1)	10 742 (25.9)	13 448 (32.4)	14 420 (34.7)
$150 000-$199 999	1928 (10.0)	6131 (31.8)	6200 (32.1)	5033 (26.1)
$200 000 and above	3984 (18.1)	8176 (37.2)	6124 (27.9)	3686 (16.8)
Did not report	2510 (4.3)	9174 (15.7)	16 360 (28.0)	30 386 (52.0)
Survey cycle				
September 14-26, 2022	3201 (5.9)	10 335 (19.2)	15 059 (28.0)	25 345 (47.0)
October 5-17, 2022	2871 (5.3)	10 349 (19.1)	15 175 (28.0)	25 844 (47.7)
November 2-14, 2022	3170 (5.8)	9977 (18.3)	15 656 (28.8)	25 615 (47.1)
December 9-19, 2022	2915 (5.5)	9963 (18.9)	14 738 (27.9)	25 240 (47.8)
January 4-16, 2023	2983 (5.6)	10 114 (19.0)	15 072 (28.3)	25 157 (47.2)
February 1-13, 2023	2816 (5.2)	10 230 (19.0)	15 335 (28.5)	25 348 (47.2)
Region				
Northeast	3330 (6.0)	11 169 (20.2)	15 863 (28.7)	24 956 (45.1)
South	6403 (5.2)	21 102 (17.1)	34 008 (27.5)	62 133 (50.3)
Midwest	3923 (5.9)	13 644 (20.1)	19 410 (29.2)	29 490 (44.4)
West	4300 (5.6)	15 054 (19.5)	21 753 (28.2)	35 970 (46.7)

^a^ Data were from the most recent 6 waves of the Household Pulse Survey (HPS) conducted in September 2022 through February 2023. Of the total of 369 328 respondents in the combined sample, 322 507 (93.2%) reported that prices for goods and services had increased in the area where they live and shop in the last 2 months. Individuals agreeing with the statements “I do not think prices have changed” (10 232 respondents), “I think prices have decreased” (2892 respondents), “I do not know” (10 586 respondents), or with data not reported or missing (24 209 respondents) were excluded from our analysis since the question about inflation stress was not asked among those respondents. Personal weights were used to produce representative population estimates.

^b^ Race and ethnicity were self-reported and coded using a combination of 2 questions in the survey: (1) “What is your race? Please select all that apply” including choices of Asian, Black, White, other, or multiple races; (2) “Are you of Hispanic, Latino, or Spanish origin?” with a binary response. Race and ethnicity were then recoded into 5 categories: Hispanic, non-Hispanic Asian, non-Hispanic Black, non-Hispanic White, and other (any other race or ethnicity alone, or race and ethnicity in combination).

In the ordinal logistic regression models of inflation stress, model 1 showed that women exhibit significantly higher inflation stress than men (OR, 1.30; 95% CI, 1.27-1.34) (Table 2). Compared with White respondents, Black (OR, 1.25; 95% CI, 1.19-1.32) and Hispanic (OR, 1.65; 95% CI, 1.57-1.74) respondents reported higher inflation stress, whereas Asian respondents reported lower inflation stress (OR, 0.86; 95% CI, 0.80-0.91). Individuals who were widowed (OR, 1.54; 95% CI, 1.44-1.64), divorced (OR, 1.57; 95% CI, 1.51-1.63), or separated (OR, 1.99; 95% CI, 1.74-2.27), but not individuals who had never been married, experienced higher inflation stress than married couples. Inflation stress was higher among middle-aged groups (eg, ages 31 to 40 years: OR, 1.11; 95% CI, 1.05-1.17) than their older and younger counterparts.

**Table 2.** Ordinal Logistic Regressions Estimating Sociodemographic Variations in Inflation Stress^a^

Characteristics	Odds ratio (95% CI)	
	Model 1 (n = 321 332)	Model 2 (n = 321 332)
Gender		
Male	1 [Reference]	1 [Reference]
Female	1.30 (1.27-1.34)	1.28 (1.24-1.31)
Race and ethnicity		
White	1 [Reference]	1 [Reference]
Asian	0.86 (0.80-0.91)	1.07 (1.00-1.14)
Black	1.25 (1.19-1.32)	1.03 (0.98-1.09)
Hispanic	1.65 (1.57-1.74)	1.26 (1.20-1.33)
Other^b^	1.47 (1.38-1.57)	1.32 (1.24-1.42)
Marital status		
Married	1 [Reference]	1 [Reference]
Widowed	1.54 (1.44-1.64)	1.01 (0.95-1.08)
Divorced	1.57 (1.51-1.63)	1.08 (1.03-1.12)
Separated	1.99 (1.74-2.27)	1.24 (1.08-1.43)
Never married	1.03 (0.99-1.07)	0.75 (0.72-0.78)
Age group		
18-20 y	1 [Reference]	1 [Reference]
31-40 y	1.11 (1.05-1.17)	1.30 (1.23-1.38)
41-50 y	1.10 (1.04-1.16)	1.31 (1.23-1.38)
51-60 y	0.98 (0.93-1.03)	1.07 (1.01-1.14)
61-70 y	0.70 (0.66-0.73)	0.63 (0.60-0.67)
≥71 y	0.49 (0.46-0.52)	0.43 (0.41-0.46)
Education		
<High school	NA	1 [Reference]
Some high school	NA	0.91 (0.73-1.14)
High school	NA	0.90 (0.74-1.10)
Some college	NA	0.80 (0.66-0.98)
Associate’s	NA	0.80 (0.65-0.98)
Bachelor’s	NA	0.51 (0.42-0.62)
Graduate	NA	0.41 (0.34-0.50)
Household income		
<$25 000	NA	1 [Reference]
$25 000-$34 999	NA	0.87 (0.80-0.94)
$35 000-$49 999	NA	0.73 (0.68-0.78)
$50 000-$74 999	NA	0.58 (0.54-0.62)
$75 000-$99 999	NA	0.44 (0.41-0.47)
$100 000-$149 999	NA	0.33 (0.31-0.35)
$150 000-$199 999	NA	0.23 (0.22-0.25)
$200 000 and above	NA	0.14 (0.13-0.15)
Did not report	NA	0.59 (0.55-0.63)
Survey cycle		
September 14-26, 2022	1 [Reference]	1 [Reference]
October 5-17, 2022	1.04 (0.99-1.10)	1.05 (0.99-1.10)
November 2-14, 2022	1.02 (0.97-1.07)	1.03 (0.98-1.08)
December 9-19, 2022	1.03 (0.99-1.08)	1.06 (1.01-1.11)
January 4-16, 2023	1.01 (0.96-1.06)	1.05 (1.00-1.10)
February 1-13, 2023	1.01 (0.97-1.06)	1.06 (1.01-1.11)
Region		
Northeast	1 [Reference]	1 [Reference]
South	1.18 (1.14-1.23)	1.06 (1.01-1.10)
Midwest	1.01 (0.97-1.05)	0.87 (0.83-0.91)
West	0.99 (0.95-1.03)	0.96 (0.92-1.01)
Estimated cut points^c^		
Cut 1	0.07 (0.07-0.08)	0.02 (0.01-0.02)
Cut 2	0.42 (0.39-0.45)	0.11 (0.09-0.13)
Cut 3	1.53 (1.42-1.63)	0.44 (0.35-0.54)

Abbreviation: NA, not applicable.

^a^ The dependent variable inflation stress is coded with 4 ordinal categories, with 1 being “not at all stressful” and 4 being “very stressful.” Model 1 included variables for gender, race and ethnicity, marital status, age group, and region as well as Household Pulse Survey wave. Model 2 added education and household income as covariates to the base model. Repeating the analysis using ordinary least squares regression yielded similar patterns.

^b^ Including multiple races.

^c^ In ordinal logistic regression, cut points refer to the thresholds that separate different levels of the ordinal outcome variable. The cut points are estimated by the model and were typically set based on the values of the dependent variable in the sample. In this case, the outcome variable is a 4-point Likert scale ranging from not at all stressful to very stressful, and the 3 cut points were the estimated values that distinguish each category of the scale (ie, 1-2, 2-3, 3-4). They were used to define the categories of the outcome variable and to calculate the probabilities of being in each category based on the estimated coefficients and the values of the independent variables.

Model 2 showed that higher education (eg, graduate degree: OR, 0.41; 95% CI, 0.34-0.50) and income levels ($200 000 and above: OR, 0.14; 95% CI, 0.13-0.15) were associated with lower inflation stress. Including these socioeconomic status (SES) indicators had little difference in the gender gap (OR, 1.28; 95% CI, 1.24-1.31), but it did result in major changes in racial differences. With adjustments, the difference for Black respondents was no longer statistically significant. Possibly due to their higher SES, Asian respondents may have experienced a lower level of inflation stress. However, after adjusting SES, Asian respondents showed slightly higher inflation stress (OR, 1.07; 95% CI, 1.00-1.14). SES also seemed to explain a significant share of differences in inflation stress across marital status. The higher levels of inflation stress among middle-aged individuals become more substantial after adjusting for SES differences (ages 31 to 40 years: OR, 1.30; 95% CI, 1.23-1.38).

## Discussion

This study found that rising inflation has become a significant source of stress, especially among women and those who were socioeconomically more vulnerable. The cross-sectional nature of the data used prevented establishing long-term causal effects. In the time of high inflation, there is an urgent need for research and policy development to safeguard public health and prevent the worsening of health disparities.^7^

## Notes

Supplement 1. **eAppendix.** Analytical Strategy

Supplement 2.
Data Sharing Statement
