# Supplementary material for: Assessment of Sociodemographics and Inflation-Related Stress in the US
Source: JAMA Netw Open. 2023 May 15;6(5):e2313431. doi: 10.1001/jamanetworkopen.2023.13431 (PMC12507453; doi:10.1001/jamanetworkopen.2023.13431)
Supplement: Supplement 1. — eAppendix. Analytical Strategy [file jamanetwopen-e2313431-s001.pdf]

# Supplemental Online Content

Wu C, Louie P, Bierman A, Schieman S. Assessment of sociodemographics and inflation-related stress in the US. *JAMA Netw Open.* 2023;6(5):e2313431. doi:10.1001/jamanetworkopen.2023.13431

**eAppendix.** Analytical Strategy

This supplemental material has been provided by the authors to give readers additional information about their work.

© 2023 Wu C et al. *JAMA Network Open.*

**eAppendix.** Analytical Strategy

The Household Pulse Survey (HPS) is an online, probability-based survey conducted by the U.S. Census Bureau to rapidly measure the social and economic effects of coronavirus and other emergent issues on American households. Data collection began on April 23, 2020, and currently follows a two-weeks on, two-week off collection and dissemination approach. Starting on September 14, 2022, the HPS added questions on inflation and changes in behaviors due to inflation. The data are publicly available through the U.S. Census Bureau webpage (https://www.census.gov/programs-surveys/household-pulse-survey/datasets.html).

In this study, we use the data from the most recent six waves of the HPS conducted in September (Sep 14-26, n=50,937), October (Oct 5-17, n=42,040), November (Nov 2-14, n=61,453), and December (Dec 9-19, n=70,685) in 2022 as well as January (Jan 4-16, n=68,504) and February (Feb 1-13, n=75,709) of 2023. Combining all six waves leads to a total of 369,328 respondents. Of the total respondents, 328,624 (93.2%) reported that prices for goods and services have increased in the area where they live and shop in the last two months. Individuals (6.8%) who indicated that "I do not think prices have changed" (n=10,451), "I think prices have decreased" (n=2,954), or "I do not know" (10,586) were excluded from our analysis since the question about inflation stress was not asked among them. Listwise deletion leads to an analytical sample of 321,332 with no missing values on all key variables for our regression analysis.

The dependent variable *inflation stress* is measured using the question asking, "How stressful, if at all, has the increase in prices in the last two months been for you?". The response includes 1= "not at all stressful", 2= "a little stressful", 3= "moderately stressful", and 4= "very

stressful”. Key socio-demographics include gender, race, marital status, and age. Gender at birth is coded in a binary manner with 0 corresponding to man, and 1 to woman. Race is self-reported including five categories: 1= “Non-Hispanic White”, 2= “Non-Hispanic Black”, 3= “Hispanic”, 4= “Non-Hispanic Asian”, and 5= “Other”. Marital status includes 1= “married”, 2= “widowed”, 3= “divorced”, 4= “separated”, and 5= “single, never married”. Age is measured in years, ranging from 18 to 88. To explore the non-linear effect of age on inflation stress, we recoded age into a categorical variable with six categories: 1= “18-30 years old”, 2= “31-40 years old”, 3= “41-50 years old”, 4= “51-60 years old”, 5= “61-70 years old”, and 6= “71 or above”. Two major socioeconomic indicators are the level of education and household income. Education includes seven categories: 1= “less than high school”, 2= “some high school”, 3= “high school graduate or equivalent”, 4= “some college, but degree not received or in progress”, 5= “associate’s degree”, 6= “bachelor’s degree”, 7= “graduate degree”. Household income includes eight categories of 1= “less than $25,000”, 2= “$25,000-$34,999”, 3= “$35,000-$49,999”, 4= “$50,000-$74,999”, 5= “$75,000-$99,999”, 6= “$100,000-$149,999”, 7= “$150,000-$199,999”, and 8= “$200,000 and above”. Two controls are the region and wave of the HPS. The region includes four categories of 1= “Northeast”, 2= “South”, 3= “Midwest”, and 4= “West”. Wave of the HPS includes six monthly categories from September 2022 to February 2023.

The analysis takes two general steps. First, we describe the distribution of inflation stress overall as well as by waves. The goal is to demonstrate that most Americans have been stressed about rising prices, and consistently for a long period (e.g., over six months). Second, we use stepwise ordered logistic regression to estimate the social distribution of inflation stress and test whether the difference in socioeconomic status may help explain some of the demographic disparities in inflation stress. The dependent variable inflation stress is coded on an ordered scale

© 2023 Wu C et al. *JAMA Network Open.*

with four categories from not at all stressful (1) to very stressful (4). We use ordered logistic regression models or specifically the cumulative odds models with the assumption that our independent variables have an equal effect on the odds of changing from one category to another. Odds ratios are used for the interpretation of the results. Specifically, the base model, Model (1) includes variables of gender, race, marital status, and age group. The region and also week of the survey are also controlled. Model (2) adds two indicators of socioeconomic status, level of education, and household income to Model (1). Comparing changes in the effects of demographic variables between the two models provides a general idea about how socioeconomic inequalities in terms of education and household income may or may not account for the demographic disparities in inflation stress. We are aware that the changes in coefficients may not be solely driven by the added SES indicators in nonlinear models. We, therefore, repeat the analysis using OLS regressions. Results show that the overall patterns are consistent. The HPS person weights are used in all analysis to produce representative population estimates.

© 2023 Wu C et al. *JAMA Network Open.*
