# Supplementary material for: Assessment of Sociodemographics and Inflation-Related Stress in the US
Source: JAMA Netw Open. 2023 May 15;6(5):e2313431. doi: 10.1001/jamanetworkopen.2023.13431 (PMC12507453; doi:10.1001/jamanetworkopen.2023.13431)
Supplement: Supplement 2. — Data Sharing Statement [file jamanetwopen-e2313431-s002.pdf]

Data Sharing Statement

Wu. Assessment of Sociodemographics and Inflation-related Stress in the US. *JAMA Netw Open*. Published May 15, 2023. doi:10.1001/jamanetworkopen.2023.13431

Data
Data available: Yes
Data types: Other (please specify)
Additional Information: The data are publicly available through the U.S. Census Bureau webpage (https://www.census.gov/programs-surveys/household-pulse-survey/datasets.html).
How to access data: HPS data https://www.census.gov/data/experimental-data-products/household-pulse-survey.html
When available: With publication

Supporting Documents
Document types: None

Additional Information
Who can access the data: The data are publicly available through the U.S. Census Bureau webpage (https://www.census.gov/programs-surveys/household-pulse-survey/datasets.html).
Types of analyses: for any purpose
Mechanisms of data availability: The data are publicly available through the U.S. Census Bureau webpage (https://www.census.gov/programs-surveys/household-pulse-survey/datasets.html).
Any additional restrictions: none
